# Mol­ecular and crystal structure of 2,5-bis­[(4-fluoro­phen­yl)imino­meth­yl]furan

**DOI:** 10.1107/S2056989025005006

**Published:** 2025-06-17

**Authors:** Moira K. Lauer, Gary J. Balaich, Scott T. Iacono, Nathan J. Weeks

**Affiliations:** a Department of Chemistry and Physics, University of North Carolina at Pembroke, Pembroke, NC 28372, USA; b Department of Chemistry & Chemistry Research Center, USAF Academy, Colorado, Springs, CO 80840, USA; Vienna University of Technology, Austria

**Keywords:** crystal structure, furan, monomer, green chemistry

## Abstract

The title furan bis­(imine) compound, 2,5-bis­[(4-fluoro­phen­yl)imino­meth­yl]furan, C_18_H_12_F_2_N_2_O, was synthesized by condensation of 2,5-furan­dicarboxaldehyde with two equivalents of 4-fluoro­aniline. The mol­ecular structure consists of a central furan ring symmetrically bound to nearly coplanar imino­methyl groups with N-bonded 4-fluoro­phenyl rings that are significantly tipped out of the plane of the furan ring. In the crystal structure, the furan ring lies on a twofold rotation axis in space group *C*2/*c* with the furan ring and imine groups of adjacent mol­ecules participating in C—H⋯N inter­actions to give furan-ring-centered hydrogen-bonded chains extending along [010]. Further cohesion of the crystal structure is achieved by participation of the peripheral 4-fluoro­phenyl rings in C—H⋯F hydrogen bonding and edge-to-face C—H⋯π inter­actions, resulting in a tri-periodic network. The resulting supra­molecular chains formed by C—H⋯F hydrogen bonding extend in a direction parallel to [101].

## Abstract 2

In the reported crystal structure, the central furan ring lies on a twofold rotation axis in space group *C*2/*c* with the furan ring and imine groups of adjacent mol­ecules participating in C—H⋯N inter­actions to give furan-ring-centered hydrogen-bonded chains extending along [010].

## 1. Chemical context

**Figure 1:**
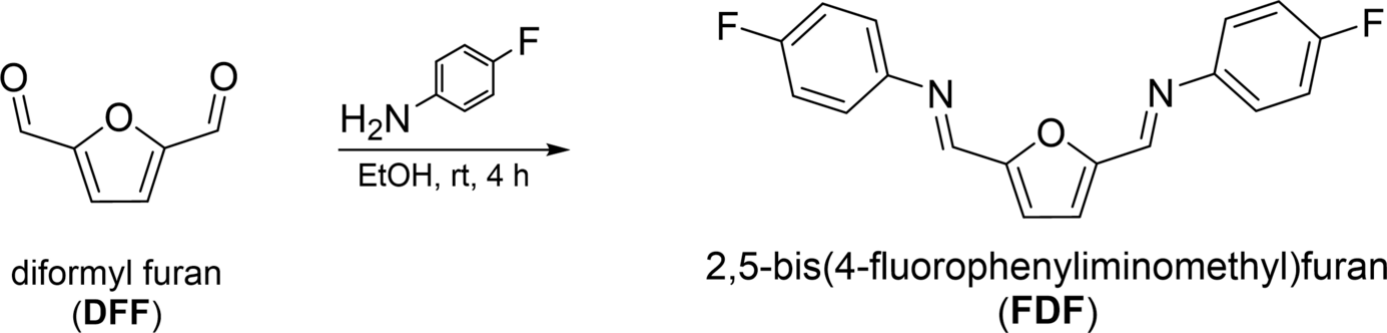
Single-step reaction of 2,5-bis­[(4-fluoro­phen­yl)imino­meth­yl]furan in the environmentally friendly solvent ethanol.

The ongoing plastic pollution crisis and limited recycling strategies related to polyethyl­ene terephthalate (PET) has led to significant research on the development of alternative materials possessing similar mechanical and gas barrier properties (Yoshida *et al.*, 2016 ▸; Thio­unn & Smith, 2020 ▸; Lauer & Smith, 2020 ▸). Polyethyl­ene furan­oate (PEF) may function as a drop-in replacement for PET plastics due to the structural similarity of the furanic core of PEF relative to the phenyl core of PET (Fei *et al.*, 2020 ▸). Di­fluoro-terminated furanic monomers have already been developed and used to synthesize thermally robust (*T_d_*-5% = 753–766 K) aryl ether ketones, but the synthesis of these monomers required the use of several deleterious reagents and yields were not published (Bao *et al.*, 2019 ▸). However, 2,5-furan­dicarboxaldehyde-derived imines can be synthesized in a single step in an environmentally friendly solvent with high yields, low energy requirements, facile isolation, and in excellent purity. This green chemistry approach was used to synthesize the title compound, 2,5-bis­[(4-fluoro­phen­yl)imino­meth­yl]furan (Fig. 1 ▸), a possible candidate for the development of next-generation bio-based polymeric materials.
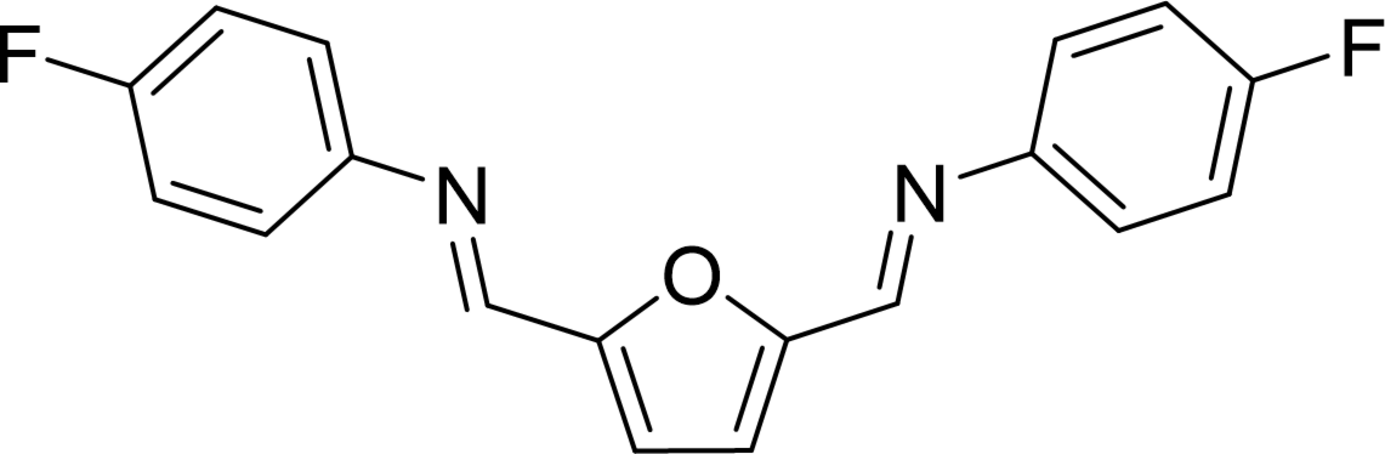


## 2. Structural commentary

**Figure 2:**
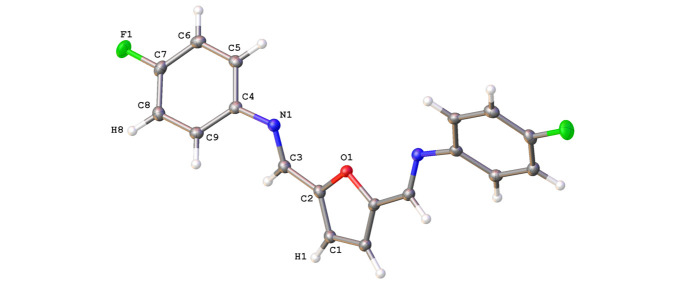
Mol­ecular structure of 2,5-bis­[(4-fluoro­phen­yl)imino­meth­yl]furan. The central furan ring lies on a twofold rotation axis in space group *C*2/*c* with the planes of the 4-fluoro­phenyl rings tipped out of the central furan ring plane by 34.38 (3)°. Displacement ellipsoids are shown at the 50% probability level, with H atoms of arbitrary size; non-labeled atoms are generated by symmetry operation −*x* + 1, *y*, −*z* + 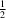.

Mol­ecules of 2,5-bis­[(4-fluoro­phen­yl)imino­meth­yl]furan crystallize in space group *C*2/*c* with one half mol­ecule per asymmetric unit. Bond lengths alternate long [C1—C1^i^ = 1.411 (2) Å; symmetry code: (i) −*x* + 1, *y*, −*z* + 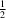] – short [C1—C2 = 1.3678 (15) Å] – long [C2—C3 = 1.4353 (15) Å], as expected for the central furan ring symmetrically bound to the C atoms of two methanimine groups (Fig. 2 ▸). The furan ring lies on a twofold rotation axis with nearly co-planar methanimine groups [small O1—C2—C3—N1 torsion angle of −3.35 (15)°] bound through their N atoms to 4-fluoro­phenyl groups as the *E* isomer in a *δ*-*cis* conformation (Fig. 2 ▸). In the crystal structure of the non-fluorinated 2,5-bis­(phenyl­imino­meth­yl)furan mol­ecule, a similar core structure was reported, with the peripheral benzene rings significantly tipped out of the plane of the central furan ring at a reported torsional angle of 38° (Mallet *et al.*, 2011 ▸). The title mol­ecule displays a similar peripheral ring tip, with the planes of the 4-fluoro­phenyl groups tipped out of the plane of the central furan ring [34.38 (3)°] as well as the plane of the methanimine groups [39.03 (11)°].

## 3. Supra­molecular features

**Figure 3:**
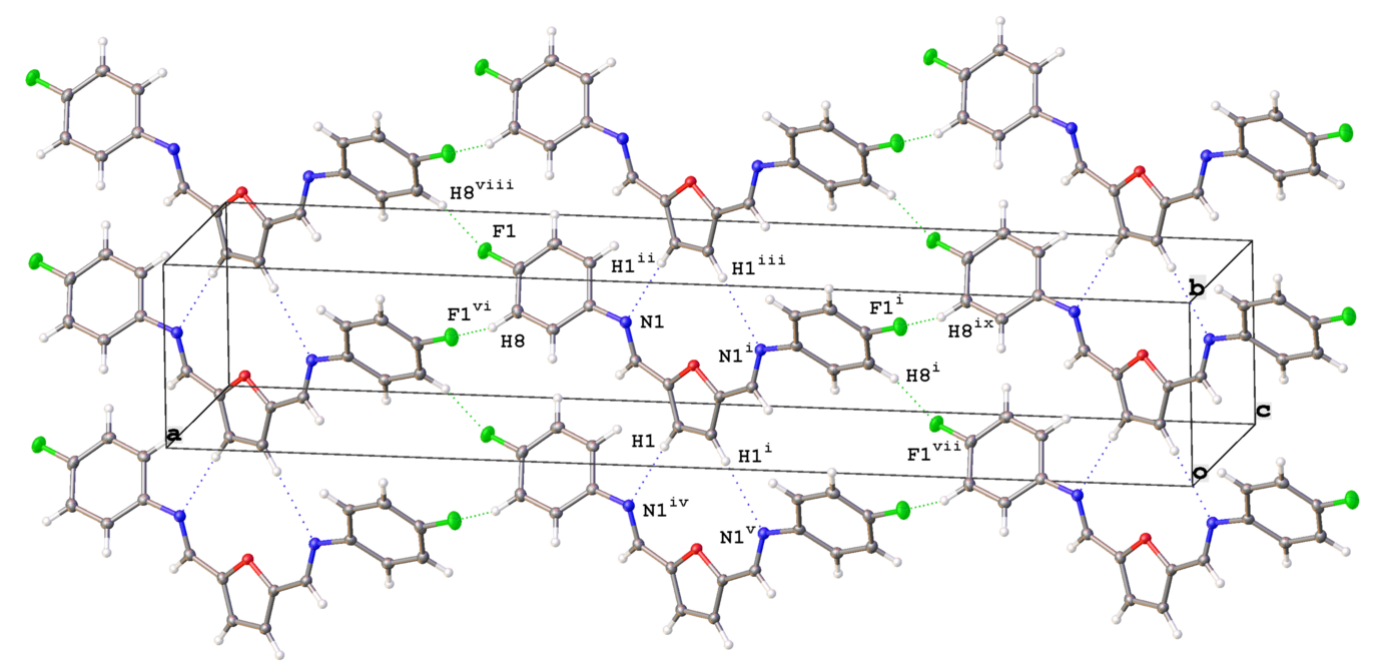
Hydrogen-bonding motif with unit cell overlay for 2,5-bis­[(4-fluoro­phen­yl)imino­meth­yl]furan. Each mol­ecule forms eight hydrogen bonds using C—H⋯N [2.576 (14) Å] and C—-H⋯F [2.617 (14) Å] inter­actions. Displacement ellipsoids are shown at the 50% probability level, with H atoms of arbitrary size. [Symmetry codes: (i) 1 − *x*, *y*, 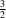 − *z*; (ii) *x*, 1 + *y*, *z*; (iii) 1 − *x*, 1 + *y*, 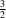 − *z*; (iv) *x*, *y* − 1, *z*; (v) 1 − *x*, *y* − 1, 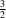 − *z*; (vi) 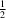 − *x*, *y* − 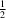, 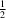 − *z*; (vii) 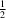 + *x*, *y* − 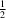, 1 + *z*; (viii) 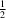 − *x*, 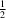 + *y*, 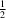 − *z*; (ix) 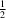 + *x*, 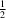 + *y*, 1 + *z*.]

**Figure 4:**
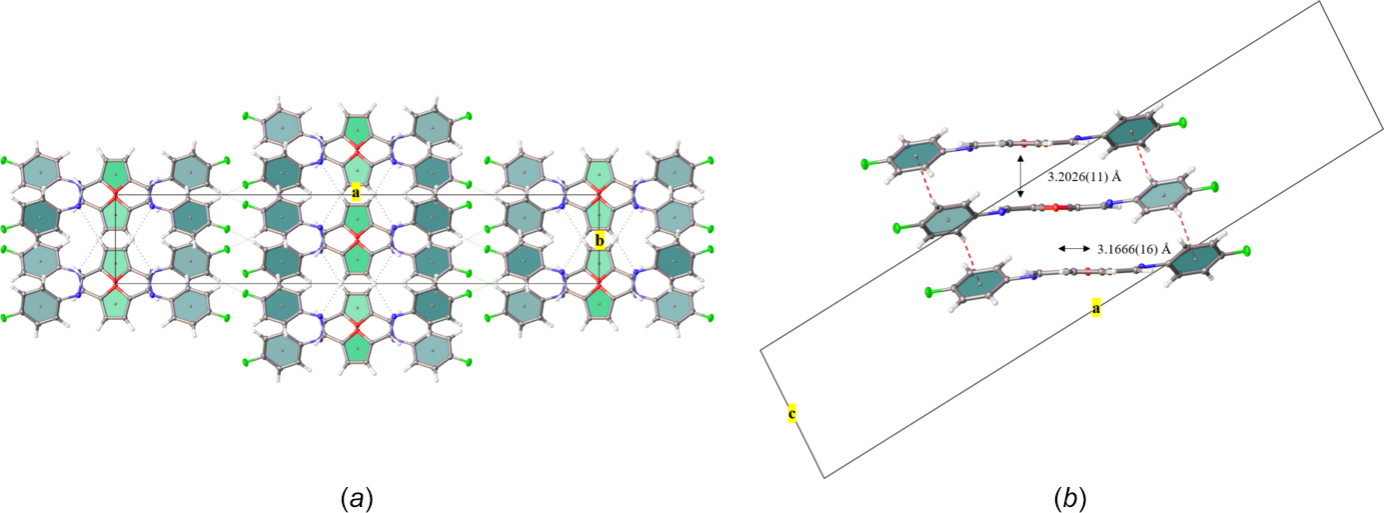
Details of the packing for 2,5-bis­[(4-fluoro­phen­yl)imino­meth­yl]furan. The view along [001] (*a*) shows the head-to-head arrangement of the central furan rings in addition to the edge-to-face inter­actions between peripheral 4-fluoro­phenyl groups. A portion of the view along [010] (*b*) depicts the offset furan ring-to-furan ring inter­planar spacing as well as the C—H⋯π inter­actions [H6⋯*Cg*2 (2.6004 (4) Å] and H9⋯*Cg*2 [2.6384 (4) Å] that extend along [001]. Displacement ellipsoids are shown at the 50% probability level, with H atoms of arbitrary size.

The crystal structure of 2,5-bis­[(4-fluoro­phen­yl)imino­meth­yl]furan is consolidated by a tri-periodic network consisting of C—H⋯N and C—H⋯F hydrogen bonds as well as weaker edge-to-face C—H⋯π inter­actions. Mol­ecules pack head (N1) to tail (C1*H*), held in place by four furan-ring-centered C1—H1⋯N1 inter­molecular hydrogen bonds [2.576 (14) Å, Table 1 ▸] per mol­ecule, forming chains that run along [010] (Fig. 3 ▸). Further, the two 4-fluoro­phenyl rings of each mol­ecule inter­act with the 4-fluoro­phenyl rings of adjacent mol­ecules, forming four additional C—H⋯F hydrogen bonds [2.617 (14) Å, Table 1 ▸] per mol­ecule that repeat in a direction parallel to [101] (Fig. 3 ▸). Adjacent mol­ecules pack along [001] in a head-to-head orientation, resulting in the O atoms of the co-parallel central furan rings facing opposite directions with an inter­plane spacing of 3.2026 (11) Å but with their centroids (*Cg*1) offset by 3.1666 (16) Å (Fig. 4 ▸). Although the planes of the 4-fluoro­phenyl rings (corresponding centroid is *Cg*2) are co-parallel along [010] (Fig. 3 ▸), they are mutually tilted at an angle of 58.35 (5)° along [001], giving edge-to-face 4-fluoro­phenyl group C—H⋯π contacts that involve H6⋯*Cg*2 [2.6004 (4) Å] and H9⋯*Cg*2 [2.6384 (4) Å inter­actions], see Fig. 4 ▸. Data for the non-fluorinated 2,5-bis­(phenyl­imino­meth­yl)furan gave a furan ring-to-furan ring inter­plane spacing of 3.3 Å with a reported C—H⋯π contact distance of 2.63 Å (Mallet *et al.*, 2011 ▸). The C—H⋯F contact distance in the crystal structure for the title mol­ecule [2.617 (14) Å, Table 1 ▸] is also consistent with the range of values reported for the perfluoro­phenyl compound, 2,5-bis­(penta­fluoro­phenyl­imino­meth­yl)furan [2.50 (4)–2.77 (5) Å; (Mallet *et al.*, 2011 ▸]. Although π–π stacking inter­actions were observed in the packing pattern of 2,5-bis­(penta­fluoro­phenyl­imino­meth­yl)furan (Mallet *et al.*, 2011 ▸), the incorporation of only one F atom on each peripheral ring in the title mol­ecule produced a mol­ecular and crystal structure that more closely resembles that of the non-fluorinated 2,5-bis­(phenyl­imino­meth­yl)furan, and that is consolidated by C—H⋯N and C—H⋯F hydrogen bonds as well as edge-to-face C—H⋯π inter­actions.

## 4. Database survey

The crystal structures of the related compounds, 2,5-bis­(phenyl­imino­meth­yl)furan [Cambridge Structural Database (CSD; Groom *et al.*, 2016 ▸) deposition identifier EBEVIS] and 2,5-bis­(penta­fluoro­phenyl­imino­meth­yl)furan (CSD deposition number EBEVUE) were previously reported (Mallet *et al.*, 2011 ▸). Compared to the title mol­ecule, 2,5-bis­(phenyl­imino­methyl­furan) crystallizes in the same space group type (*C*2/*c*) with similar unit cell parameters (Mallet *et al.*, 2011 ▸), giving nearly identical mol­ecular and crystal structures. However, 2,5-bis­(penta­fluoro­phenyl­imino­meth­yl)furan crystallizes in space group *P*1, having a mol­ecular structure with one methanimine arm in the *δ-cis* conformation and the other arm in the *δ-trans* conformation in addition to a packing pattern featuring π—π stacking inter­actions (Mallet *et al.*, 2011 ▸). The consolidating effect of C—H⋯N hydrogen bonding was not discussed for the reported crystal structures of 2,5-bis­(phenyl­imino­meth­yl)furan and 2,5-bis­(penta­fluoro­phenyl­imino­meth­yl)furan, but C—H⋯F inter­actions were noted for the structure of 2,5-bis­(penta­fluoro­phenyl­imino­meth­yl)furan (Mallet *et al.*, 2011 ▸).

## 5. Synthesis and crystallization

To a well-stirred solution of 2,5-furan­dicarboxaldehyde (0.200 g, 1.6 mmol) in ethanol (20 ml) was added 4-fluoro­aniline (0.394 g, 3.5 mmol), and the reaction mixture heated to 313 K. The reaction mixture was allowed to stir until all of the monosubstituted product had converted to the disubstituted product as determined by GC–MS. The reaction mixture was allowed to cool, diluted by half with water, filtered, and washed with water. After drying at 333 K under reduced pressure, a greenish-yellow-colored crystalline solid was obtained (0.402 g, 80%).

## 6. Refinement

Crystal data, data collection and structure refinement details are summarized in Table 2 ▸. All hydrogen atoms, except H1 and H8, were placed using a riding model with their positions constrained relative to their parent C atom using the appropriate HFIX command in *SHELXL* (Sheldrick, 2015*b* ▸). Hydrogen atoms H1 and H8 involved in C—H⋯N and C—H⋯F hydrogen bonding were placed from the electron-density map, and their C—H distances restrained (DFIX, C—H range 0.94–0.96 Å) at 0.95 Å with *U*_iso_(H) = 1.2*U*_eq_(C).

## Supplementary Material

Crystal structure: contains datablock(s) I. DOI: 10.1107/S2056989025005006/wm5760sup1.cif

Supporting information file. DOI: 10.1107/S2056989025005006/wm5760Isup3.mol

Supporting information file. DOI: 10.1107/S2056989025005006/wm5760sup4.docx

HKL. DOI: 10.1107/S2056989025005006/wm5760sup5.txt

Supporting information file. DOI: 10.1107/S2056989025005006/wm5760Isup5.cml

CCDC reference: 2455881

Additional supporting information:  crystallographic information; 3D view; checkCIF report

## Figures and Tables

**Table 1:** Hydrogen-bond geometry (Å, °)

*D*—H⋯*A*	*D*—H	H⋯*A*	*D*⋯*A*	*D*—H⋯*A*
C1—H1⋯N1^i^	0.970 (14)	2.576 (14)	3.5408 (14)	173.1 (10)
C8—H8⋯F1^ii^	0.945 (14)	2.617 (14)	3.2815 (13)	127.8 (10)

Symmetry codes: (i) 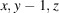; (ii) 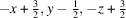.

**Table 2:** Experimental details

Crystal data	
Chemical formula	C_18_H_12_F_2_N_2_O
*M*_r_	310.30
Crystal system, space group	Monoclinic, *C*2/*c*
Temperature (K)	100
*a*, *b*, *c* (Å)	32.9033 (3), 6.02694 (5), 7.14998 (6)
β (°)	95.5021 (8)
*V* (Å^3^)	1411.35 (2)
*Z*	4
Radiation type	Cu *K*α
μ (mm^−1^)	0.93
Crystal size (mm)	0.21 × 0.11 × 0.07

Data collection	
Diffractometer	XtaLAB Synergy, Dualflex, HyPix3000
Absorption correction	Gaussian (*CrysAlis PRO*; Rigaku OD, 2023 ▸)
*T*_min_, *T*_max_	0.671, 1.000
No. of measured, independent and observed [*I* > 2σ(*I*)] reflections	13137, 1312, 1252
*R*_int_	0.028
(sin θ/λ)_max_ (Å^−1^)	0.605

Refinement	
*R*[*F*^2^ > 2σ(*F*^2^)], *wR*(*F*^2^), *S*	0.029, 0.078, 1.05
No. of reflections	1312
No. of parameters	114
H-atom treatment	H atoms treated by a mixture of independent and constrained refinement
Δρ_max_, Δρ_min_ (e Å^−3^)	0.20, −0.17

Computer programs: *CrysAlis PRO* (Rigaku OD, 2023 ▸), *SHELXT* (Sheldrick, 2015*a* ▸), *SHELXL* (Sheldrick, 2015*b* ▸) and *OLEX2* (Dolomanov *et al.*, 2009 ▸).

## supplementary crystallographic information

### 2,5-Bis[(4-fluorophenyl)iminomethyl]furan Crystal data

**Table:**

C_18_H_12_F_2_N_2_O	*F*(000) = 640
*M**_r_* = 310.30	*D*_x_ = 1.460 Mg m^−^^3^
Monoclinic, *C*2/*c*	Cu *K*α radiation, λ = 1.54184 Å
*a* = 32.9033 (3) Å	Cell parameters from 10588 reflections
*b* = 6.02694 (5) Å	θ = 2.7–68.8°
*c* = 7.14998 (6) Å	µ = 0.93 mm^−^^1^
β = 95.5021 (8)°	*T* = 100 K
*V* = 1411.35 (2) Å^3^	Rectangular prism, clear greenish yellow
*Z* = 4	0.21 × 0.11 × 0.07 mm

### 2,5-Bis[(4-fluorophenyl)iminomethyl]furan Data collection

**Table:**

XtaLAB Synergy, Dualflex, HyPix3000 diffractometer	1312 independent reflections
Radiation source: micro-focus sealed X-ray tube, PhotonJet (Cu) X-ray Source	1252 reflections with *I* > 2σ(*I*)
Mirror monochromator	*R*_int_ = 0.028
Detector resolution: 10.0000 pixels mm^-1^	θ_max_ = 68.9°, θ_min_ = 2.7°
ω scans	*h* = −31→39
Absorption correction: gaussian (CrysAlisPro; Rigaku OD, 2023)	*k* = −7→7
*T*_min_ = 0.671, *T*_max_ = 1.000	*l* = −8→8
13137 measured reflections	

### 2,5-Bis[(4-fluorophenyl)iminomethyl]furan Refinement

**Table:**

Refinement on *F*^2^	Hydrogen site location: mixed
Least-squares matrix: full	H atoms treated by a mixture of independent and constrained refinement
*R*[*F*^2^ > 2σ(*F*^2^)] = 0.029	*w* = 1/[σ^2^(*F*_o_^2^) + (0.0395*P*)^2^ + 1.169*P*] where *P* = (*F*_o_^2^ + 2*F*_c_^2^)/3
*wR*(*F*^2^) = 0.078	(Δ/σ)_max_ = 0.001
*S* = 1.05	Δρ_max_ = 0.20 e Å^−^^3^
1312 reflections	Δρ_min_ = −0.17 e Å^−^^3^
114 parameters	Extinction correction: SHELXL-2019/2 (Sheldrick 2015b), Fc^*^=kFc[1+0.001xFc^2^λ^3^/sin(2θ)]^-1/4^
0 restraints	Extinction coefficient: 0.00100 (16)
Primary atom site location: dual	

### 2,5-Bis[(4-fluorophenyl)iminomethyl]furan Special details

**Table:**

Geometry. All esds (except the esd in the dihedral angle between two l.s. planes) are estimated using the full covariance matrix. The cell esds are taken into account individually in the estimation of esds in distances, angles and torsion angles; correlations between esds in cell parameters are only used when they are defined by crystal symmetry. An approximate (isotropic) treatment of cell esds is used for estimating esds involving l.s. planes.

### 2,5-Bis[(4-fluorophenyl)iminomethyl]furan Fractional atomic coordinates and isotropic or equivalent isotropic displacement parameters (Å^2^)

**Table:**

	*x*	*y*	*z*	*U*_iso_*/*U*_eq_	
C1	0.51924 (3)	0.11462 (17)	0.30286 (14)	0.0162 (3)	
F1	0.73109 (2)	0.88737 (12)	0.71765 (9)	0.0280 (2)	
N1	0.57648 (3)	0.62961 (15)	0.42481 (12)	0.0158 (2)	
O1	0.500000	0.46983 (17)	0.250000	0.0150 (3)	
C2	0.52992 (3)	0.33229 (18)	0.33073 (14)	0.0152 (2)	
C3	0.56719 (3)	0.42300 (18)	0.41997 (14)	0.0160 (2)	
H3	0.586445	0.322231	0.480032	0.019*	
C4	0.61637 (3)	0.68551 (17)	0.50427 (14)	0.0149 (2)	
C5	0.62174 (3)	0.88471 (17)	0.60383 (14)	0.0165 (3)	
H5	0.598685	0.974298	0.621945	0.020*	
C6	0.66028 (3)	0.95307 (19)	0.67642 (14)	0.0185 (3)	
H6	0.663913	1.088192	0.744385	0.022*	
C7	0.69331 (3)	0.81989 (19)	0.64753 (15)	0.0189 (3)	
C8	0.68949 (3)	0.62270 (19)	0.54995 (15)	0.0180 (3)	
C9	0.65075 (3)	0.55601 (18)	0.47783 (14)	0.0163 (3)	
H9	0.647485	0.420854	0.409678	0.020*	
H1	0.5356 (4)	−0.012 (2)	0.3467 (17)	0.019 (3)*	
H8	0.7127 (4)	0.537 (2)	0.5300 (19)	0.027 (4)*	

### 2,5-Bis[(4-fluorophenyl)iminomethyl]furan Atomic displacement parameters (Å^2^)

**Table:**

	*U*^11^	*U*^22^	*U*^33^	*U*^12^	*U*^13^	*U*^23^
C1	0.0153 (6)	0.0160 (5)	0.0175 (5)	0.0018 (4)	0.0024 (4)	0.0015 (4)
F1	0.0172 (4)	0.0342 (4)	0.0314 (4)	−0.0070 (3)	−0.0038 (3)	−0.0062 (3)
N1	0.0146 (5)	0.0164 (5)	0.0162 (4)	0.0003 (3)	0.0004 (3)	−0.0007 (3)
O1	0.0130 (5)	0.0137 (5)	0.0179 (5)	0.000	−0.0010 (4)	0.000
C2	0.0138 (5)	0.0168 (5)	0.0149 (5)	0.0023 (4)	0.0011 (4)	0.0014 (4)
C3	0.0153 (5)	0.0165 (5)	0.0160 (5)	0.0021 (4)	0.0011 (4)	0.0013 (4)
C4	0.0165 (5)	0.0146 (5)	0.0133 (5)	−0.0007 (4)	0.0004 (4)	0.0030 (4)
C5	0.0191 (6)	0.0143 (5)	0.0162 (5)	0.0016 (4)	0.0022 (4)	0.0025 (4)
C6	0.0240 (6)	0.0161 (5)	0.0155 (5)	−0.0030 (4)	0.0019 (4)	−0.0004 (4)
C7	0.0156 (5)	0.0236 (6)	0.0169 (5)	−0.0053 (4)	−0.0012 (4)	0.0022 (4)
C8	0.0161 (6)	0.0203 (6)	0.0177 (5)	0.0021 (4)	0.0021 (4)	0.0019 (4)
C9	0.0187 (5)	0.0152 (5)	0.0149 (5)	−0.0001 (4)	0.0009 (4)	0.0007 (4)

### 2,5-Bis[(4-fluorophenyl)iminomethyl]furan Geometric parameters (Å, º)

**Table:**

C1—C1^i^	1.411 (2)	C4—C5	1.3983 (15)
C1—C2	1.3678 (15)	C4—C9	1.4020 (15)
C1—H1	0.970 (14)	C5—H5	0.9500
F1—C7	1.3573 (12)	C5—C6	1.3861 (15)
N1—C3	1.2819 (14)	C6—H6	0.9500
N1—C4	1.4198 (13)	C6—C7	1.3825 (16)
O1—C2^i^	1.3712 (12)	C7—C8	1.3778 (16)
O1—C2	1.3712 (12)	C8—C9	1.3880 (15)
C2—C3	1.4353 (15)	C8—H8	0.945 (14)
C3—H3	0.9500	C9—H9	0.9500
			
C1^i^—C1—H1	128.1 (8)	C6—C5—C4	120.82 (10)
C2—C1—C1^i^	106.43 (6)	C6—C5—H5	119.6
C2—C1—H1	125.5 (8)	C5—C6—H6	120.8
C3—N1—C4	116.75 (9)	C7—C6—C5	118.40 (10)
C2^i^—O1—C2	105.61 (12)	C7—C6—H6	120.8
C1—C2—O1	110.76 (10)	F1—C7—C6	118.43 (10)
C1—C2—C3	128.81 (10)	F1—C7—C8	118.77 (10)
O1—C2—C3	120.34 (10)	C8—C7—C6	122.79 (10)
N1—C3—C2	124.98 (10)	C7—C8—C9	118.29 (10)
N1—C3—H3	117.5	C7—C8—H8	120.8 (9)
C2—C3—H3	117.5	C9—C8—H8	120.9 (9)
C5—C4—N1	118.31 (9)	C4—C9—H9	119.6
C5—C4—C9	118.84 (10)	C8—C9—C4	120.86 (10)
C9—C4—N1	122.74 (10)	C8—C9—H9	119.6
C4—C5—H5	119.6		
			
C1^i^—C1—C2—O1	0.69 (14)	C3—N1—C4—C9	37.67 (14)
C1^i^—C1—C2—C3	−175.88 (10)	C4—N1—C3—C2	−174.20 (9)
C1—C2—C3—N1	172.93 (11)	C4—C5—C6—C7	0.19 (15)
F1—C7—C8—C9	−179.59 (9)	C5—C4—C9—C8	0.43 (15)
N1—C4—C5—C6	−176.78 (9)	C5—C6—C7—F1	179.63 (9)
N1—C4—C9—C8	176.64 (9)	C5—C6—C7—C8	−0.02 (17)
O1—C2—C3—N1	−3.35 (15)	C6—C7—C8—C9	0.05 (17)
C2^i^—O1—C2—C1	−0.27 (6)	C7—C8—C9—C4	−0.26 (16)
C2^i^—O1—C2—C3	176.63 (11)	C9—C4—C5—C6	−0.39 (15)
C3—N1—C4—C5	−146.10 (10)		

Symmetry code: (i) −*x*+1, *y*, −*z*+1/2.

### 2,5-Bis[(4-fluorophenyl)iminomethyl]furan Hydrogen-bond geometry (Å, º)

**Table:**

*D*—H···*A*	*D*—H	H···*A*	*D*···*A*	*D*—H···*A*
C1—H1···N1^ii^	0.970 (14)	2.576 (14)	3.5408 (14)	173.1 (10)
C8—H8···F1^iii^	0.945 (14)	2.617 (14)	3.2815 (13)	127.8 (10)

Symmetry codes: (ii) *x*, *y*−1, *z*; (iii) −*x*+3/2, *y*−1/2, −*z*+3/2.
