# Supplementary material for: Mol­ecular and crystal structure of 2,5-bis­[(4-fluoro­phen­yl)imino­meth­yl]furan
Source: Acta Crystallogr E Crystallogr Commun. 2025 Jun 17;81(Pt 7):623–6. doi: 10.1107/S2056989025005006 (PMC12230620; doi:10.1107/S2056989025005006)
Supplement: Supplementary file 3 [file e-81-00623-sup4.docx]

**Synthesis:**

Diformylfuran (**DFF**, Ambeed, 0.199 g, 1.60 mmol, 1.0 eq) was dissolved in 10 mL of ethanol. 4-fluoroaniline (Themo Scientific, 0.393 g, 3.54 mmol, 2.2 eq) was added to the solution and it was allowed to stir at room temperature for several hours. The solution was diluted with 10 mL of water and filtered. Isolated yield: 0.416 g, 83.5%. The product could also be prepared with in-house synthesized DFF from fructose.

^1^H NMR (CDCl_3_, 500 MHz, δ [ppm]): δ 8.36–8.46 (s, 2H), δ 7.23–7.31 (t, 4H), δ 7.13–7.19 (s, 2H), δ 7.07–7.15 (t, 4H).

MS (EI, 70 eV): m/z (% relative intensity) 310 (M^+^, 100), 95 (C_6_H_4_F, 57), 122 (C_6_H_6_N_2_O, 32), 188 (C_11_H_12_N_2_O, 21), 311 (M+1, 20).

**Instrumentation:**

Infrared spectra were collected on a Thermo Scientific Nicolet iS50 FT-IR with an ATR attachment. Spectra were collected from 400 to 4000 cm^−1^ in transmittance mode with a resolution of 4. Fifty scans were collected. Differential scanning calorimetry was conducted on a TA DSC2500 under a flow of nitrogen gas at 50 mL∙min^−1^. Samples weighing approximately 5 mg were put into sealed aluminum crucibles. Runs were conducted by premelting to 180 °C at 10 °C∙min^−1^ and holding isothermally for 4 minutes. Two full cooling and heating cycles were then conducted from −60 °C to 180 °C. Thermogravimetric analysis was conducted on a TA TGA5500 under a flow of nitrogen gas at 50 mL∙min^−1^. Samples weighing approximately 10 mg were placed into platinum pans and heated from 20–800 °C at 10 °C∙min^−1^. Nuclear magnetic resonance was conducted on an on a Jeol (500 MHz) spectrometer. All samples were dissolved in CDCl_3_. GC-MS was conducted on an Agilent 7890A GC with an Agilent 5975C with triple-axis MS detector. Runs were conducted under a flow of argon gas at 1 mL∙min^−1^. Injections of 2 μL with a split ratio of 10 were injected with an initial 2 min temperature hold at 80 °C, then a temperature gradient of 80 to 250 °C at 15 °C∙min^−1^. Detection was accomplished with a refractive index detector. The mass spectrometry electron ionization source was set to 230 °C and the detector to 150 °C.

**Figure S1.** ^1^H NMR spectrum of 2,5-bis(4-fluorophenyliminomethyl)furan in CDCl_3_. The residual solvent signal overlaps with the signal for H_B_.

**A)**

**B)**

**Figure S2.** Stacked FTIR spectra of diformyl furan (**DFF**, black trace) and 2,5-bis(4-fluorophenyliminomethyl)furan (**FDF**, blue trace) showing either A) the full spectrum from 400–4000 cm^−1^ or B) The spectrum from 400–1700 cm^−1^. Characteristic aldehydic signals are observed exclusively for **DFF** at 2878 and 2750 cm^−1^ (doublet, C–H stretching), 1663 cm^−1^ (C=O stretching), and 1410 cm^−1^ (C–H bending). A signal characteristic of the imine in **FDF** is observed at 1623 cm^−1^ (C=N stretching). A new signal for C–F stretching in FDF is observed at 1296 cm^−1^. Several new signals for the aromatic group in **FDF** are observed form C–C stretching in the aromatic ring (1589 and 1543 cm ^−1^) and C–H aromatic out of plane bending (750, 717, and 673 cm^−1^).

**Figure S3.** TGA mass loss (black trace) and derivative (blue trace) curves of 2,5-bis(4-fluorophenyliminomethyl)furan. The *T*_d_ (5%) can be observed at 194 °C.

**Figure S4.** DSC heating (black trace) and cooling (blue trace) curves taken from the second heat-cool cycle for 2,5-bis(4-fluorophenyliminomethyl)furan. A melting endotherm can be observed at 170 °C (131 J/g) and a crystallization exotherm can be observed at 87 °C.

**M^+^**

310

311

122

95

188

**Figure S5.** Mass spectrum of 2,5-bis(4-fluorophenyliminomethyl)furan. The molecular ion peak can be observed at 310 m/z.
